# The Structure of a Daily Food Ration of the Inhabitants Over 40 Years Old in the Republic of Kazakhstan

**Published:** 2018-08

**Authors:** Roza SHAKIYEVA, Aigul ABDULDAYEVA, Kamshat AKHMETOVA, Gulnar TULESHOVA, Kulsara DOSMAMBETOVA, Nuriliya MALTABAROVA, Tatyana V. KIM, Saltanat URAZOVA, Madina GATAUOVA, Akzhunus OREKESHEVA, Altyn MOLDABAYEVA, Nazym GALIMGOZHINA, Aigul AINABAI, Madina SHARIPOVA

**Affiliations:** 1. Kazakh Academy of Nutrition, Almaty, Kazakhstan; 2. JSC “Astana Medical University”, Astana, Kazakhstan

## Dear Editor-in-Chief

We carried out a selective cross-section research of actual nutrition of the inhabitant of the Republic of the age category over 40 yr old. The subjects of the research from 2011 – 2016 were the persons over the 40 yr old (1916 people). Field research took place in 13 administrative territories (11 regions and 2 cities of Almaty and Astana). The actual feeding was assessed by the method of a 24-h recall. According to the results of questionnaire there was production a calculation of quantity of consumed food on single methodic on prescription sheets are given to each interviewer. For the calculation of chemical composition of the rations, there was used database developed by Kazakh Academy of Nutrition (KAN). At assessment of adequacy of feeding for referent volume, there were taken the normative of WHO also normative of micronutrients composition on the scale FAO/WHO (1).

At the assessment of nutritional status of the population of the republic over 40 yr old the disturbances of food were revealed in 76.4% of the cases in women, in 68.1% in men. Malnutrition was estimated in 1.1% of women, excessive in 75.3%, absence of disturbance of nutritional status in 23.6% of women. In men these volumes made up accordingly 1.8%, 66.2%, 32.0%.

The respondents with excessive body mass over 25 kgr/m^2^ were 1446 people. Among the examined 1961 people for the obesity of different degree, there were diagnosed 696 (35.5%) people. In calculations of chemical composition of ration (Table 1) caloric value of nutrition turned out to be at the level of 1603.9±642.3 kcal for women, 1839.0±727.2 kcal for men. The actual consumption of the protein became equal to 39.2–40.5 gr/1000 kcal, reference indications of FAO/WHO suppose on the protein the density not lower than the rations 25–30 gr/1000 kcal/fats of vegetable origin made up in the ration 43.4%-42.1% from the total consumption of fat. The saturated fat acids supplied in women 10.5% and in men 10.2% out of total caloric value of feeding in recommended quota less than 10% of a daily caloric value. On food density the consumption of USFA also exceeded the normative a little bit, 11.7–11.3 gr/1000 kcal at necessary level<11.0 gr basically, because of consumption of high quota of animal products by the inhabitants.

**Table 1:** Actual level of in taking of basic food substances and energy by respondents

***Nutrition factors***	***Women***		***Men***	
	***Mean***	***SD***	***Mean***	***SD***
1	2	3	4	5
Energy	1603.9	642.3	1839.0	727.2
Protein	62.8	28.3	74.4	35.0
Animal protein	37.8	23.3	44.1	29.3
Fats	60.6	30.6	65.5	34.8
Vegetable fats	26.3	18.6	27.6	21.6
USFA	18.7	11.5	20.8	12.1
MUSFA	17.5	9.5	19.8	9.9
PUSFA	13.1	9.6	14.4	10.7
Carbohydrates	197.9	92.1	229.1	98.8
Starch	120.2	60.7	156.2	70.4
Plain carbohydrates	82.3	56.5	79.3	53.3
Food fibers	15.5	7.6	17.0	7.2
Cellulose	5.7	3.6	5.6	3.4
Potassium	2681.1	1252.0	2835.8	1319.1
Calcium	528.9	308.2	549.6	353.5
Iodine	41.2	32.6	44.1	30.5
Vitamin D	0.92	1.71	1.15	2.20
Thiamine (B1)	0.731	0.314	0.845	0.396
Pantothenic acid	3.06	1.77	3.50	2.00
Biotin	15.94	13.52	17.57	13.00

The correlation of PUSFA/USFA in food was 0.9 of relative units in normative of WHO within the volume of 0.5–0.9 units.

- The contribution of the plain carbohydrates in the daily caloric value 20.5%-17.2% by twofold exceeding the normative of WHO equal to 10% out of a daily caloric value;
- Low level of food fiber consumption – 15.5–17.0 gr/per day is recommended for these groups of the inhabitant 20–25 gr/per day; on food density at necessary level 14gr/1000 kcal in women, 9.2 gr/1000 kcal in men.
- Calcium entering of calcium with food was in women 528.9±308.2 mg/per day, in men 549.6±353.5 mfr/per day at need of 1200 mg/per day; the portion of the persons with the deficiency of alimentary calcium came up to equal among the women 80.6%, among the men 80.1%; on food density the consumption of calcium came up to equal with 329.9 mg/1000 kcal in women and 298.9 mg/1000 kcal in men;
- The portion of the people with the deficiency of consumption of alimentary vitamin D came up to among the women 98.4%, among the men 96.5%, on food density to 1000 kcal at necessary level 2.5–5.0 mg consumption came up to 0.62 mg in women, 0.64 mg in men;
- Average daily consumption of iodine by the women was 42.2±30.5 mkgr/day in need of 150 mkgr/day. According to the food density to 1000 kcal at necessary level of 75vmkgr consumption it came up to 25.7 mkgr in women, 24.0 mkgr in men.
- Average daily consumption of potassium was in women 2681.1±1252.0 mg/day, in men 2835.8±1319.1 mg/day; these values are lower than recommended volumes of FAO/WHO 3.5 mhr/day;
- Deficiency of vitamin B1 – average daily intake was 0.73±0.31 mg/day in women, in need of 1.1 mg/day and 0.85±0.4 mg/day at need of 1.2 mg/day in men; on food density to 1000 kcal at necessary level 0.5–0.8 mg intake was 0.46 mg;
- Deficiency of biotin – average daily intake was 15.94±13.52 mkgr/day in women,17.57±13.0 mkgr/day in men in need of 30 mkgr/day;
- Deficiency of pantothenic acid – average daily intake was 3.06±1.77 mg/day in women, 3.50±2.0 mg/day in men in need of 5.0 mg/day. According to the result, the supply of the inhabitants of the country over 40 yr old with many vitally important food substances was adequate. Moreover, in actual condition of nutrition in the groups of inhabitant of mature, elderly and senile age there is high level of consumption of plain carbohydrates, low level of consumption of food fibers and polymicronutrient insufficiency of nutritional vitamins and mineral elements.
